# Coroners and Practitioners

**Published:** 1896-01-25

**Authors:** 


### Coroners and Practitioners.

It is a favourite doctrine with many medical men that their sole duty is to their patients, and that the outside public has nothing to do with their proceedings. There is one point, however, in which the most private of private practitioners becomes a public official and must be careful to conform to the law of the land, viz., when he certifies the cause of death. It behoves every medical man then very carefully to study the requirements of the Act which governs this proceeding, not merely because by any irregularity he may defeat the objects for which the Act was passed, but for the much more personal reason that carelessness in the giving of certificates may lead him into difficulties with officers of the law, and may readily suggest to a censorious world unpleasant suspicions, not easily thrown off, of complicity with fraud or crime.

Mr. Braxton Hicks, the well-known coroner, has written a short pamphlet, entitled "Hints to Medical Practitioners Concerning the Granting of Certificates of Death," which may usefully be read by all who have this duty thrown upon them. It is much to be wished that medical men would bear in mind the true office of the coroner, which is to inquire how, when, and by what means a person came to his death. If this were always remembered, and if it were much more fully recognised than it is that reference to the coroner does not cast any slur either on the doctor or on the friends of the deceased, much needless friction would be saved. In fact, one of the main points which Mr. Braxton Hicks wishes to enforce is that not merely when there is suspicion of foul play, but whenever there is doubt of any kind, even of diagnosis, the case becomes one for the coroner. The moral of the pamphlet is, "In cases of doubt come to your coroner."

Besides this, however, it is well to make clear that while the medical man is bound under certain circumstances to give a medical certificate of the cause of death, he is equally bound to do it "to the best of his knowledge and belief."

In the first place, then, he is not bound to give a certificate unless he has attended the deceased during the last illness; and secondly, he must only certify to what has come within his own knowledge and belief. So that if he does not know he must not certify. Strictly speaking, any certificate founded upon matters or information not within his personal knowledge is unjustifiable, and no medical man should, out of regard to himself, be persuaded to give such a certificate, for there is a proper legal way of dealing with such cases, and that is by reporting them to the coroner.

In the case of deaths which, to the knowledge of the medical man, are purely natural, his duty is simple; but even here it is important that he should remember that it is his duty to give the "primary" as well as the "secondary" cause of death. This is far too often overlooked, and may easily lead to serious misunderstandings. There are certain causes of death which are peculiarly apt to be associated with accidents or injuries, such, for example, as pyæmia, erysipelas, or peritonitis. Any one of these, given as the sole cause of death, would be liable to lead the registrar to refuse a burial certificate and to refer the case to the coroner, thus putting everyone to unnecessary trouble when the cause of death is a natural one; while, on the other hand, if the doctor knew that the primary cause was unnatural, he would be guilty of a *suppressio veri* if he omitted to say so. In every case then the primary cause should be entered, and if it is not known it should be definitely stated, "Primary not known." For instance, a woman may die of peritonitis. That may be set up in consequence of some unlawful operation; and if a medical man be called in and is subsequently asked to give a certificate, then, if he is unable to account for the matter in a natural way, he would not be justified in giving a certificate of "peritonitis" simply.

We commend this useful and suggestive little pamphlet to everyone engaged in practice; but, at the same time, we cannot hide from ourselves the fact that if it were acted on up to the letter, the work of the coroner's office would be enormously increased, for the coroner would practically become the public certifier of he cause of death.

